# Wild and Domestic Canids and Their Interactions in the Transmission Cycles of *Trypanosoma Cruzi* and *Leishmania* spp. in an Area of the Brazilian Cerrado

**DOI:** 10.3390/pathogens9100818

**Published:** 2020-10-06

**Authors:** Elida M. V. Brandão, Samanta C. C. Xavier, Fabiana L. Rocha, Caio F. M. Lima, Ísis Z. Candeias, Frederico G. Lemos, Fernanda C. Azevedo, Ana M. Jansen, André L. R. Roque

**Affiliations:** 1 Laboratório de Biologia de Tripanosomatídeos, Instituto Oswaldo Cruz, Rio de Janeiro, RJ 21040-900, Brazil; elida_millena@hotmail.com (E.M.V.B.); samanta@ioc.fiocruz.br (S.C.C.X.); jansen@ioc.fiocruz.br (A.M.J.); 2 Centro de Ciências Aplicadas e Educação, Campus IV Litoral Norte, Universidade Federal da Paraíba, Rio Tinto, PB 58297-000, Brazil; lopesrocha.fabiana@gmail.com; 3 Programa de Conservação Mamíferos do Cerrado, Catalão, GO 75704-020, Brazil; mvcaiomotta@gmail.com (C.F.M.L.); izdc@hotmail.com (Í.Z.C.); lemos.pcmc@gmail.com (F.G.L.); cavalcantifer@yahoo.com (F.C.A.); 4 Fundação Parque Zoológico de São Paulo, São Paulo, SP 04301-905, Brazil; 5 Departamento de Reprodução Animal, Faculdade de Medicina Veterinária e Zootecnia, Universidade de São Paulo, São Paulo, SP 05508-270, Brazil; 6 Departamento de Ciências Biológicas, Universidade Federal de Catalão, Catalão, GO 75704-020, Brazil

**Keywords:** agroecosystems, Brazilian Cerrado, *Cerdocyon thous*, *Lycalopex vetulus*, *Chrysocyon brachyurus*, *Canis lupus familiaris*, *Trypanosoma cruzi*, *Leishmania* spp., Neotropical mammals

## Abstract

*Trypanosoma cruzi* and *Leishmania* spp. are parasites that infect multiple hosts including canids, considered bioaccumulators of parasites. Deforestation in the Cerrado biome has resulted in the exposure of wild canids to anthropized areas, where they may establish ecological and epidemiological relationships with domestic dogs. We evaluated the infection by trypanosomatids in canids from a Cerrado agroecosystem between 2013 and 2017. Samples of wild canids (blood, bone marrow and skin) and dogs (blood) were collected for parasitological, serological and molecular diagnosis. A total of 414 samples from wild (*n* = 131) and domestic (*n* = 283) canids were collected, including recaptures. We obtained five positive hemocultures from *Lycalopex vetulus* (*n* = 2), *Cerdocyon thous* (*n* = 1) and dogs (*n* = 2), all characterized as *T. cruzi* TcIII/V (18S rDNA) and TcIII/V/VI (gGAPDH); one positive skin fragment for *Leishmania* sp. (*C. thous*), one positive skin culture (*Chrysocyon brachyurus*) and one positive fresh blood examination from a dog. Infection by *T. cruzi* and *Leishmania* spp. was serologically confirmed in 18% and 4% of the canids, respectively. Active transmission was attested by seroconversion events and occurred despite the low rate of positive parasitological assays. Wild and domestic canids infected by both parasites were detected sharing the same areas, pointing to a possible spillover of parasites among them.

## 1. Introduction

Vertebrate trypanosomatid parasites are transmitted by hematophagous invertebrates, and among the different genera included in the Trypanosomatidae family, *Trypanosoma* and *Leishmania* are the most studied due to their public health importance [1]. Most of these species are described as mandatory heteroxenic; presenting enzootic or zoonotic life cycles and being transmitted, respectively, by insect vectors from orders Hemiptera (Reduviidae, Triatominae) and Diptera (Psychodidae, Phebotominae). *Trypanosoma* and *Leishmania* are parasites characterized by high genetic, biochemical and molecular diversity, which partly explains their expressive biological plasticity, which until now represents an open question. *Trypanosoma cruzi* and at least the most prevalent *Leishmania* species from Brazil (*Leishmania infantum, L. braziliensis* and *L. amazonensis)* are defined as multi-host parasites due to their ability to infect hundreds of mammalian species in the wild [2,3]. They have complex life cycles, often modulated by the trophic relationships of the associated taxa.

Neotropical wild canids comprise poorly studied taxa in terms of their possible role in the transmission cycle of *T. cruzi* and *Leishmania* spp. in nature. Six canid species occur in different Brazilian biomes: the short-eared dog *Atelocynus microtis*, the bush dog *Speothos venaticus,* the maned-wolf *Chrysocyon brachyurus,* the crab-eating fox *Cerdocyon thous*, the pampas fox *Lycalopex gymnocercus* and the hoary fox *Lycalopex vetulus* [4]. Except for *A. microtis* and *L. gymnocercus*, the other species are widely observed in the Brazilian savannah biome, the Cerrado, with *L. vetulus* being an endemic species of this ecosystem [5,6]. Most of them are omnivorous, including in their diet a wide variety of items of animal origins and fruits, both vertebrate and invertebrate [5,7,8,9]. Occupying high levels in the trophic net, for preying on mammals, lizards, snakes, frogs and insects, wild canids can be considered bioaccumulators of parasites, especially those that may be orally transmitted such as *T. cruzi.* Members of the Canidae family may be long-lived and may maintain the infection by trypanosomatid parasites during their whole life, being considered excellent study models for following up natural infections [10].

Despite the great richness and wide distribution of wild canids in the Americas, there are few studies on Trypanosomatid infection in these animals. Canids are hosts of at least two genera of trypanosomatids in the Americas: *Trypanosoma* and *Leishmania* [2,3]. Wild canids infected by *T. cruzi* were observed by serological tests [10], and parasite isolation has already been registered for hoary foxes [10,11]. The possible vector-borne transmission of *T. cruzi* among maned wolves and *Leishmania* sp. infection of several mammal taxa maintained in a Brazilian zoo were recently reported [12]. Still concerning infection by *Leishmania* spp., four wild canid species have already been found serologically infected, and *L. infantum* isolation was obtained in one bush dog and *L. infantum* and *L. amazonensis* in crab-eating foxes [13,14,15]. Domestic dogs *Canis lupus familiaris* are recognized as the main reservoir of *L. infantum*, although they have also been found infected by at least six other *Leishmania* species [16]. Its role as a *T. cruzi* reservoir varies in different regions: may be important reservoirs in the Argentine Chaco, Venezuela and the southern United States [17,18,19] or present itself infected (serologically positive), but rarely being infectious to the vector, as occurs in Brazil. In this case, dogs have been important as sentinels of the parasitosis [20].

In general, wild canid species are known to explore large areas and different habitats [4], important aspects for the dispersion of multi-host parasites. These traits, together with the growing reduction of natural habitats, have been pressing wildlife to increasingly use human-altered landscapes, where animals may cohabit more intimately with domestic dogs, favoring ecological and epidemiological relations, including parasite’s spillover [21,22]. This is a common scenario in the Brazilian Cerrado, an ecosystem that has been suffering high negative anthropic impact, leading, in some cases, to the establishment of agroecosystems [23,24]. Agroecosystems consist of three intermingled and strongly interacting sub-systems: the managed fields, referred to as the productive sub-system; the semi-natural or natural habitats surrounding them and the human sub-system composed of settlements and infrastructures [24]. In some areas of Central Brazil, as in the municipality of Cumari, Goiás, a region such as Limoeiro has about 75% or more of the original vegetation already transformed by any sort of human activity for production, especially exotic pastures (*Urochloa* sp.) for cattle. However, it is not known to what extent this modification and closer contact between wild and domestic canids impact parasite transmission.

Since 2008, wild canids in this area have been captured and monitored as part of a long-term research and conservation program [25]. Individuals of hoary foxes, crab-eating foxes and maned wolves have been diagnosed positive for trypanosomatid infection [10]. Serological tests demonstrated that the three canid species present in Limoeiro region were infected by *T. cruzi* and/or *Leishmania* spp., and *T. cruzi* DTU (Discrete Typing Unit) TcIII was also isolated from two *L. vetulus* [10,11]. In this region, domestic dogs are owned by the cowboys who live on the farms and these animals stay loose and use the area in a manner usually unknown by their owners [26]. Thus, domestic dogs and wild canids share the same areas and reports of agonistic encounters are not exceptional [27,28]. In this paper, we conducted a longitudinal study to evaluate the rate and pattern of infection by trypanosomatids in wild and domestic canids that co-inhabit the same agroecosystem, discussing the consequences of the spatial overlapping for transmission cycles.

## 2. Results

One hundred and eleven wild canids of the species *C. thous* (*N* = 72), *L. vetulus* (*N* = 29) and *C. brachyurus* (*N* = 10) were captured between 2013 and 2017. During the same period, there were 20 recaptures (15%), including four animals that were recaptured more than once, totaling 131 sampling events (Table 1).

Samples from 187 different domestic dogs were collected between 2014 and 2016. A total of 96 dogs were evaluated in 2014, 88 in 2015 and 99 in 2016, totaling 283 sampling events. From those, 94 sampling events (33%) were performed in 66 dogs that were previously evaluated, including 28 of them that were sampled in the three study-years (Table 1). There was a replacement rate of 59% from 2014 to 2015 and 36% for the following period.

### 2.1. Trypanosomatid Infection

We obtained five positive hemocultures, all of them characterized as *T. cruzi,* derived from *L. vetulus* (*N* = 2), *C. thous* (*N* = 1) and *C. l. familiaris* (*N* = 2), as shown in Table 2. One skin culture from a *C. brachyurus* (LBT 11484), captured in 2014, was positive for flagellates, but the culture was not established, and the parasite characterization was not possible. Other skin fragment preserved in ethanol from a *C. thous* (LBT 11465), captured in 2013, was positive for *Leishmania* spp. kDNA (kinetoplast deoxyribonucleic acid), but negative in all the reactions performed targeting HSP70 (Heat Shock 70 protein) and ITS1 (Internal transcribed spacer region 1) primers. No bone marrow culture or fresh blood examination from wild canids was positive. Only one fresh blood examination from a domestic dog (LBT 7163), captured in 2014, was positive for flagellates, but the hemoculture was negative.

**Figure 1:**
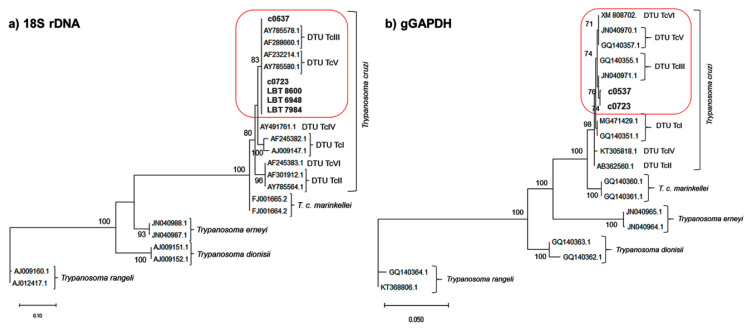
Phylogenetic analysis of (**a**) 18S rDNA (small subunit ribosomal gene) and (**b**) gGAPDH (glycosomal glyceraldehyde-3-phosphate dehydrogenase gene) sequences from positive hemocultures from wild and domestic canids captured in the Limoeiro region, Cumari, Goiás, Brazil. Red boxes were used to highlight our sequences grouped with the reference sequences identified as TcIII/V DTUs for 18S and TcIII/V/VI for gGAPDH, reinforcing that it is not possible to separate the hybrid genotypes through the molecular targets used. Three of them are sequences from culture sediment (parasite DNA from cultures that were positive at some point, but that did not establish: LBTs 8600; 6948 and 7984) and 2 (c0537 → LBT 11,477 and c0723 → LBT 7202) are pure cultures, available at Coltryp (Coleção de Trypanosoma de Mamíferos Silvestres, Domésticos e Vetores). The culture sediments did not amplify in the gGAPDH Polymerase Chain Reaction (PCR).

All samples that were positive in hemocultures were identified as *T. cruzi* DTU TcIII/TcV by the 18S rDNA gene. Of these samples, amplification by the gGAPDH target was possible for only two of them, corresponding to cultures with parasite isolation (PP) and were characterized as *T. cruzi* DTUs TcIII/V/VI (Table 2). This may be because gGAPDH is present in a lower number of copies in the genome than the 18S target, which would decrease the chances of amplification. Because these molecular targets are not able to separate hybrid genotypes from their parental ones, the DTUs involved are grouped in the same branch of the phylogenetic tree (Figure 1). We did not observe double peaks on the electropherogram of our sequences, indicative of co-infection, and after all alignment procedures, no significant differences in nucleotides that could clarify the genotype(s) involved were observed. However, as cellular cloning or Whole Genome Sequence was not performed, we cannot rule out the possibility of hybridization event in these samples, or even co-infection with different *T. cruzi* DTUs.

The tree was inferred by maximum likelihood using the Kimura 2-parameter model with a gamma-distributed rate of variation among sites (K2P + G) for 18S rDNA (= K80 + G, nomenclature used by the phyML program) and the General Time Reversible model of substitution with invariant sites (GTR + I) for gGAPDH, with bootstrapping at 1000 replicates.

### 2.2. Serological Diagnosis of Trypanosoma cruzi and Leishmania spp.

Twenty-five (32%) *C. thous* and twenty (47.6%) *L. vetulus* were positive for *T. cruzi* and/or *Leishmania* spp., either in simple or mixed infections. Two (18.2%) *C. brachyurus* were positive only for *T. cruzi* (Table 3). One of the maned wolves (LBT 11484), which had positive skin culture, was serologically positive for *T. cruzi,* with a serological titer (Indirect Immunofluorescence Antibody Test (IFAT)) of 1/160. *L. vetulus* was the species that presented largest number of positive individuals (especially for *T. cruzi*), and *C. thous* was the species with higher serological infection rates for *Leishmania* sp. infection. Forty-two (14.8%) domestic dogs were also positive for *T. cruzi* and/or *Leishmania* spp. in simple or mixed infections (Table 3).

All canids (wild and domestic) positive on parasitological and/or molecular assays were also positive on serological tests, except by one *C. thous* that was positive in the *Leishmania* kDNA-PCR in skin sample, but negative on serological tests. Most *T. cruzi*-positive canids had serological titers at the cut-off point, whereas most *Leishmania* spp. infections presented a titer of 1/80. Among them, domestic dogs presented higher titers for *T. cruzi*, ranging from 1/40 to 1/2560, while *C. thous* presented higher for *Leishmania* spp., ranging from 1/40 to 1/320 (Table 3). Of the total of canids infected with *Leishmania* spp., four of them were diagnosed as *L. infantum*, due to the positivity in the TR DPP^®^ (Rapid Test for Diagnosis of Canine Visceral Leishmaniasis, BioManguinhos, Rio de Janeiro, Brazil)) test: one *C. thous* and three domestic dogs.

The year 2014 was the only in which seropositivity was not observed for *Leishmania* spp. infection in wild canids (Table 4). Considering the expeditions to collect samples from domestic dogs, 2015 was the year in which the highest number of dogs seropositivity for *Leishmania* spp. infection (*N* = 6) was observed, although the majority was mixed infection with *T. cruzi* (*N* = 5) (Table 4).

Of the 10 recaptured *L. vetulus*, five remained positive (*Leishmania* spp. (*N* = 1) and *T. cruzi* (*N* = 4)), three seroconverted for *T. cruzi* infection, and two remained negative. Of the four hoary foxes that remained seropositive for *T. cruzi* throughout recaptures, one had been diagnosed as a mixed infection (1/160 and 1/80 for *T. cruzi* and *Leishmania* spp., respectively), but the following year maintained seropositivity only for *T. cruzi*, with the same serological titer.

Of five *C. thous* recaptured, two remained positive for *T. cruzi* (one of them presented as a mixed infection, with a titer of 1/40 for both parasites, and the following year remained positive only for *T. cruzi* (1/80)), one individual seroconverted for *Leishmania* spp. infection and two remained negatives. The only maned wolf recaptured remained seronegative (Table 4).

Of 66 resampled domestic dogs, serum samples were obtained from 61. From these, seven remained positive for *T. cruzi* since first collection (among them, one that presented as a mixed infection with a 1/40 serological titer for both parasites in 2015, in the following year it maintained only *T. cruzi* infection with an equal titer), two seroconverted for *T. cruzi* infection, and 52 remained negative for both parasites (Table 4).

**Figure 2:**
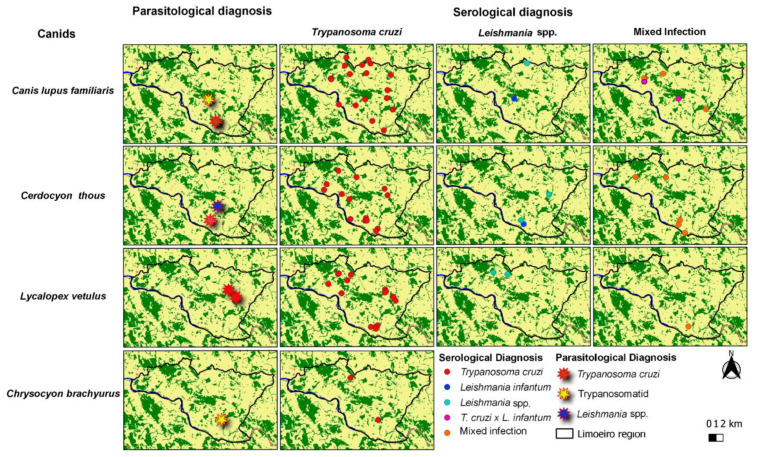
Spatial distribution of wild and domestic canids infected by *Trypanosoma cruzi, Leishmania* spp. and mixed infections, highlighting the positive parasitological diagnosis. Maps were separated by canids species (wild and domestic) captured from 2013 to 2017 in the Limoeiro region, Cumari, Goiás, Brazil. The Google Terra.cn Normal background was obtained through the QuickMapServices Plugin in Quantum GIS version 3.12.

Overall, 414 capture events (including recaptures) of wild and domestic canids succeeded. It eight positive parasitological exams were obtained, representing 1.9% of parasitological prevalence (5 wild canids and 3 domestic dogs). Seroprevalence was 21.5% (*N* = 89), including seven of the eight canids positive in the parasitological tests. Thus, the total infection rate was 21.7% (*n* = 90). The spatial distribution of infected wild and domestic canids is shown in Figure 2.

## 3. Discussion

Canids have a combination of ecological characteristics that favor opportunities to become infected with *T. cruzi* and *Leishmania* spp.: They have a diverse diet, including insects and small mammals (which may be infected by these parasites) [4] and are able to maintain different *T. cruzi* genotypes and infection by different *Leishmania* species [11,29,30]. Moreover, their wide home ranges and the use of diverse habitats [4] turn them into potential hubs of parasite dispersion. Both *T. cruzi* and *Leishmania* spp. infect wild and domestic canids at Limoeiro region, besides small mammals [31], and maintenance of these parasites by canids occurs despite the low rate of positive parasitological assays. Canids probably become infected through their exposure and consumption of infected vectors and prey. Positive hemocultures were observed in only 1.7% (*N* = 5/298) of the examined canids, and only one dog was positive for fresh blood examination, demonstrating low parasitemia and, consequently, low potential to these hosts to be source of *T. cruzi* infection vectors.

Concerning *Leishmania* sp. infection, only one *C. thous* was positive in the skin PCR, but this infection could not be further confirmed when other molecular targets (HSP70 and ITS1) were employed. This was probably because there are many more copies of the kDNA in the genome in comparison to the other targets, increasing the chances of amplification. Thus, most of the diagnosis was based on serological diagnosis. These results demonstrate that the *Leishmania* spp. infection in canids in the area, although occurring (as observed in the seropositive individuals, including an event of seroconversion) is probably associated with short periods of higher parasitism, which was not detected in any of the investigated canids during the five-year follow-up.

One skin culture of *C. brachyurus* was also positive for flagellates, but the culture was not established (and the parasite not characterized). This was one of the two maned-wolves serologically positive for *T. cruzi* infection, presenting a high serological title (1/160). This result leads us to hypothesize that the animal could be infected with *T. cruzi*, and that the detected parasites could have come from microvessels when collecting the skin, although a mixed infection with other trypanosomatid species detected in the culture cannot be rejected.

The infection by both parasites was detected in all canid species from the study area (except *Leishmania* sp. in *C. brachyurus*) and parasitemia, essential for *T. cruzi* transmission (and attested by positive hemocultures), was observed in domestic dogs, *C. thous* and *L. vetulus.* The seroconversion events observed for both parasitosis attest that the transmission was occurring in the area during the study. The overall seroprevalence of *T. cruzi* was about four times higher than the observed for *Leishmania* infection; moreover, the majority of canids infected by *Leishmania* was mixed infected with *T. cruzi.*

*C. thous, L. vetulus* and *C. brachyurus* occur simpatrically in the Cerrado [32,33] and, at Limoeiro region, these wild canids are also sintopic to domestic dogs, a factor that may influence the transmission of these parasites. Our study area fits into the concept of agroecosystem, where the relationships between wild, domestic and human animals occur with greater proximity [26]. Therefore, wild and domestic species interact more closely with each other, establishing a network of interconnections, through range overlap and interspecific contact [25], and/or participating in the food chain (i.e., consumption of small mammals and invertebrates) [9].

Animals infected with *T. cruzi* and *Leishmania* spp. were captured in the same points in the study area, which demonstrates the overlapping of these parasites’ infection. The overlap of infections at these points can lead to putative spillover of parasites between wild and domestic canids. Both *T cruzi* and *Leishmania* spp. infect wildlife and domestic canids from Limoeiro region, and infection rates were higher in wild canids and small mammals than in dogs [31]. The wider spatial distribution of *T. cruzi* is probably a consequence of the higher infection rate by this parasite in all studied mammals.

Due to the elevated population replacement rate, it was extremely difficult to monitor the infection rate of domestic dogs over the years; only 28 of the 187 examined dogs (15%) remained and were evaluated in the three years. High replacement rates were previously described for rural dog populations in Chile, Indonesia, and South Africa [34,35].

In the studied region, dogs were present in all the households and are frequently related to house and poultry protection, besides cattle herding [26]. Sampled dogs were classified as “rural free-ranging dogs” following Vanak and Gompper [36], and there were no feral dogs in the study area. It means that they were owned or peripherally associated with human habitations but were not confined to a proscribed outdoor area or kennel. Therefore, they can move freely through the landscape, favoring possible contact with wild canids and vectors, which may trigger spillover events. However, although free-ranging dogs can cover large areas, they usually spend most of their time around their residence [37,38], which could explain why dogs’ infection rates were lower than those of wild canids.

The *T. cruzi* infection pattern displayed by wild and domestic canids dogs from Limoeiro region were similar to that observed in other areas of Brazil: positive serology, indicating their exposure to the parasite’s transmission cycle, and undetectable parasitemia, demonstrated by the rarity of positive hemocultures or fresh blood examination [20]. Positive hemocultures were detected in dogs, *L. vetulus* and *C. thous,* and it has been shown that these animals share at least one DTU of *T. cruzi* (TcIII) [10]. Although we were not able to distinguish the genotype/s involved (TcIII/TcV), previous studies using RFLP-PCR in samples from this two hoary foxes confirmed the infection by DTU TcIII [11], a genotype also found in marsupials (*Gracilinanus agilis*) from the same area [31]. Considering the pattern observed for experimentally infected domestic dogs, someone can expect that both wild and domestic canids from the area present a short period of patent parasitemia during the initial phase of infection (rarely demonstrated by positive hemocultures in this study), followed by a later phase with undetectable parasitemia, even in reinfections [39].

In agreement with the study of Rocha et al. [10], we demonstrated that the transmission of *T. cruzi* is well-established in the area, with 19% of infected canids (32% considering only wild canids). In addition, for the first time we detected *T. cruzi* infection in maned wolves in this area. Infection rates were higher in wild canids, followed by wild small mammals and domestic dogs [31]. The rate of infection by *T. cruzi* in a specific host is driven by contact processes vector–parasite–environment–host; thus, we might expect that these ecological dissimilarities lead to different infection ratios [10].

Among wild canids, the infection rates observed for *L. vetulus* were about two times higher than observed for the other species, and three seroconversion events were observed. This canid was the one in which *T. cruzi* was isolated for the first time in the area [10] and represents two of the five positive hemocultures in the present study. The hoary fox seems to be most likely exposed to triatomine bugs due its habit of regularly using armadillo burrows [5,6,40], a recognized ecotope for triatomine vectors, such as species of the genus *Panstrongylus* [41]. Besides, the insectivorous diet of hoary foxes may also contribute to this higher infection rate, and the consumption of triatomine bug was already reported in the area [9].

In contrast, despite the plasticity of *C. thous* regarding habitat use, this species explores less microhabitats suitable for triatomines than hoary foxes [25]. However, *C. thous* present a diet rich on animal origin items, such as vertebrates and invertebrates [9], which could explain the infection by both *T. cruzi* and *Leishmania* spp. *T. cruzi* is recognized as a parasite that can be orally transmitted (prey-predator) [10], but amastigotes of *Leishmania* species are also capable of infecting mammalian cells (experimentally) and oral transmission cannot be ruled out in nature [42]. Maned wolves were found infected only by *T cruzi* and was the species with lowest infection rate among studied canids. Although generalists, consuming both mammals, birds and insects [8], maned wolves are the most herbivorous among the three wild canids in Limoeiro [9] consuming high rates of fruits, and this could explain their low infection rates.

Surprisingly, considering the already described importance of dogs and wild canids (specially *C. thous)* as reservoirs of *L. infantum* [13,16,43], less than 5% of the canids were infected by *Leishmania* spp. (8.4% considering only wild canids). We demonstrated infection by *L. infantum* (confirmed by TR DPP^®^ test) in *C. thous* and domestic dogs, representing less than 1% of the examined animals. In fact, *C. thous* populations were already reported as dependent to the contact with domestic dogs to maintain the transmission cycle [44]. Even though the rate of *Leishmania* species infection among the dogs in the area was low, still *C. thous* and dogs were the most infected canid species. The high density and the absence of movement restraint of dogs are factors that would increase the chance of encounter between them, pointing to the importance of overlapping transmission to sustain the *Leishmania* transmission [44]. Even so, *Leishmania* transmission was observed in the area since the first evaluation (as observed in the positive kDNA-PCR in *C. thous*) and later confirmed by the seropositive mammals, including seroconversion. Two aspects should be considered: (i) all reactions were performed using an anti-dog IgG (not species-specific for wild canids) and, because of that, are less sensitive for antibody detection, which can result in the sub-estimation of real infection rates in these canid species; and (ii) despite the cautious criteria adopted for serological diagnosis, the overlapping of transmission cycles favors the natural co-infection of wild and domestic canids with different species of trypanosomatids and cross-diagnosis are always prone to occur.

Canids appear to behave as selective filters of parasite species/genotypes (controlling and maintaining infection at low parasitism levels) when compared to small mammals, where greater richness of trypanosomatids was detected [31]. Interactions and competitive exclusion between tripanosomatid species and/or *T. cruzi* subpopulations certainly take a role in modulating infection ratios [10].

Knowing that wild and domestic canids in the Limoeiro region share trypanosomatid infections, this enzootic scenario has to be analyzed from a space-time perspective, in order to understand the displacements and contact rates between canids in the area, as well as possible landscape factors that may be correlated. This may provide a better understanding of parasite’s spillover processes and dispersion in agroecosystems and, ultimately, of the potential risk of infection for local human communities.

## 4. Materials and Methods

### 4.1. Study Area

The study area comprises private cattle farms of Limoeiro region, municipality of Cumari (18°22.02′ S, 48°5.48′ W), southeast of Goiás State, Brazil. Although inserted on an ecotone area between two ecosystems, the Atlantic Forest and the Cerrado, the vast majority of the area (75%) has been altered by human activities and is mostly covered by exotic pasture (*Urochloa* sp.). The remaining is represented by fragments of original vegetation, such as gallery and semideciduous forests [25]. Climate in the region has two well-marked seasons, one hot and wet and another cold and dry [45].

### 4.2. Canids Capture and Sample Collection

**Figure 3:**
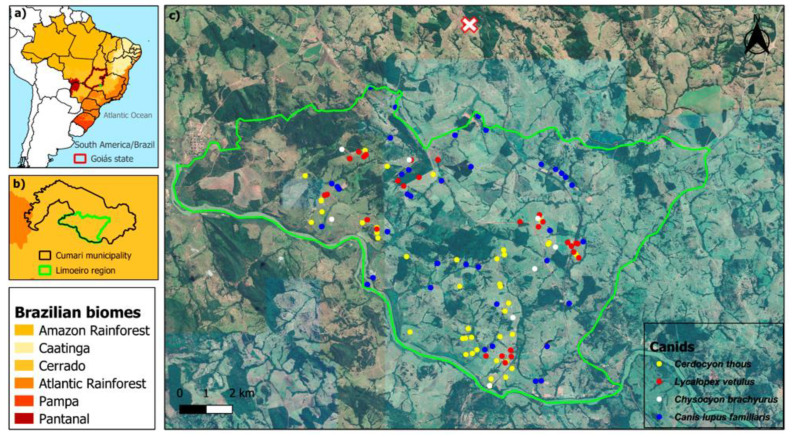
Geographical location of the study area: (**a**) Southeast of the Goiás state, Brazil; (**b**) municipality of Cumari, Limoeiro region, and (**c**) spatial distribution of capture points of wild and domestic canids, between 2013 and 2017. An individual of *Lycalopex vetulus* was captured 3,7km from Limoeiro region, shown by the symbol “
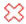
”.

Canids were captured in annual campaigns of 30 to 60 days each, from 2013 to 2017 for wild canids, and from 2014 to 2016 for domestic canids. Wild canids were captured using box traps made with galvanized wire mesh baited with sardines (for the capture of *C. thous* and *L. vetulus*) or with a mixture of sardines and cooked chicken (for the capture of *C. brachyurus*). Traps were distributed and inspected every morning on each expedition at locations where animals were viewed through night-time focusing (adapted from [46]) and registered through camera-traps and/or tracks and signs (Figure 3).

Wild canids were anesthetized with an association of 15 mg/kg of ketamine (Cetamin 10 mg/mL, Syntec, Santana de Parnaiba, SP, Brazil), 0.5 mg/kg of midazolam (Dormire 5mg/mL, Cristália Chemical and Pharmaceutical Products, São Paulo, SP, Brazil) and 0.2 mg/kg butorphanol (Torbugesic 10 mg/mL, Fort Dodge Laboratories, Fort Dodge, IA, USA.) administered intramuscularly in a single injection into the gluteal muscle.

**Figure 4:**
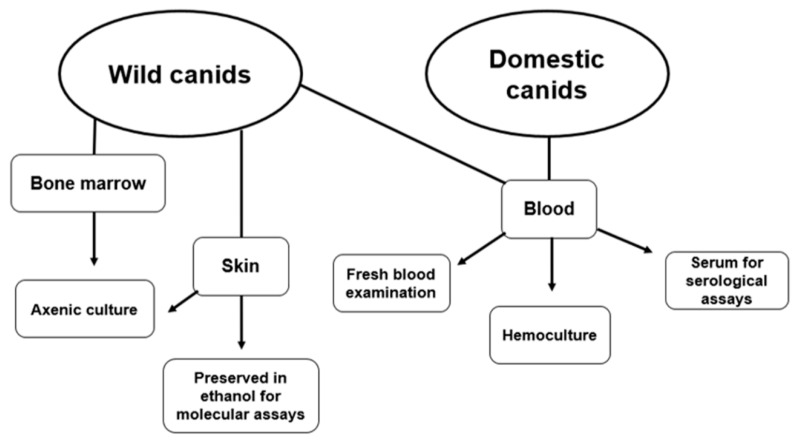
Scheme showing the biological materials collected for respective parasitological, serological and molecular assays from wild and domestic canids in the Limoeiro region, Cumari, Goiás, Brazil.

All captured wild canids were marked with ear-tags and subcutaneous microchip for individual identification. We collected blood samples by puncture of the brachial cephalic vein using Vacutainer^®^ tubes with anticoagulant (Ethylenediamine Tetraacetic Acid (EDTA)) for fresh blood examination and hemoculture and EDTA-free tubes that were centrifugated for serum separation and then stocked at −20 °C until serology assay. Aspiration of bone marrow by puncture of the iliac crest using 40 × 12 mm needles and 10 mL syringe was also performed, then transferred to Vacutainer^®^ tubes with anticoagulant for subsequent inoculation in culture medium. In addition, intact skin biopsies were obtained from two sites in the iliac crest region, using a 3-mm punch. We transferred these fragments to tubes containing: (i) sterile saline (sodium chloride (NaCl) at 58.44 g/mol) with antibiotic and antifungal (10 mg streptomycin, 25 μL amphotericin B and 10,000 IU penicillin per mL, Sigma^®^ commercial solution, San Luis, MO, USA) to later inoculation in culture tubes and (ii) absolute ethanol for molecular diagnosis (Figure 4). After total recovery from anesthesia, animals were released at the site of the capture.

Domestic dogs were actively searched on all farms in the study area (Figure 3), and an individual questionnaire was also applied after formal authorization and concordance of the owner. They were individually identified by microchips and were physically restrained with help of owners. From these animals, only blood samples were collected, following the same procedures above described for wild canids (Figure 4).

### 4.3. Parasitological and Molecular Diagnosis

The fresh blood examination was carried out with a drop of approximately 5 µL of blood between slide and coverslip in an optical microscope at 400× magnification to visualize flagellates. Hemocultures were performed by the inoculation of 0.6–0.8 mL of blood divided into two tubes containing biphasic NNN (Novy-Mc Neal-Nicole) medium with LIT (Liver Infusion Tryptose) and examined every two weeks up to five months [10].

Bone marrow was transferred from Vacutainer^®^ tubes (Franklin Lakes, NJ, USA) with EDTA to one or two tubes containing NNN culture medium and Schneider’s medium supplemented with 10% fetal bovine serum as liquid phase. Skin fragments collected in saline tubes were stored for 24h at 4 °C and then transferred to culture tubes containing NNN medium and Schneider liquid medium [47]. In both cases, the cultures were examined every four days up to two months.

Isolated parasites were amplified, cryopreserved, and deposited in the Coleção de *Trypanosoma* de Mamíferos Silvestres, Domésticos e Vetores, COLTRYP/Fiocruz (Oswaldo Cruz Foundation, Rio de Janeiro, RJ, Brazil). For molecular characterization, isolated parasites were washed with 1 mL of PBS, pH 7.2, (Phosphate Buffer Solution) and centrifuged at 448 g for 10 min. The supernatant was discarded, and the pellet was stored at −20 °C until DNA extraction. The PP (Parasite Pellet) was incubated with Proteinase K, and DNA extraction was performed using QIAamp DNA Blood minikit (Qiagen, Hilden, Germany) following manufacturer’s instructions.

Positive cultures that did not result in parasite amplification for isolation (cultures not established) were directly centrifuged, and the sediments were stored at −20 °C until DNA extraction. The CP (Culture Pellet) was incubated with proteinase K and SDS (Sodium Dodecyl Sulfate), and genomic DNA was extracted with the standard phenol-chloroform method [48].

Skin fragments collected in absolute ethanol were re-hydrated with Nuclease-free water, and the DNA extraction was carried out using the Wizard Genomic DNA Purification Kit (Promega, Madison, WI, USA) according to the manufacturer’s recommendations. Aiming to detect *Leishmania* sp. infection, kDNA-PCR were performed using the pureTaq Ready-To-Go PCR beads (Amersham Biosciences, Buckinghamshire, UK) and primers directed to the conserved region of the *Leishmania*-kDNA mini circle: forward: 5′-GGGAGGGGCGTTCTGCGAA-3′ and reverse: 5′-GGCCCACTATATTACACCAACCCC-3′ [49,50].

Positive and negative controls were derived from spleen and liver fragments from infected (*Leishmania braziliensis*—IOC-L2483) and non-infected hamsters provided by the animal facilities of the Oswaldo Cruz Foundation. The PCR products were visualized after electrophoresis on 8% polyacrylamide gel and silver stained using a specific kit (DNA Silver Staining, GE Healthcare, Chicago, IL, USA). We considered positive for *Leishmania* sp. infection the presence of DNA amplified products of 120 to 140bp. One skin sample that reacted positive in kDNA-PCR was submitted to other molecular targets aiming the *Leishmania* species characterization.

#### Molecular Characterization of Positive Samples

Molecular characterization was performed for positive hemocultures and one positive skin sample in *Leishmania* sp. kDNA-PCR. Two distinct molecular reactions were employed in positive hemocultures (PP and CP): 18S SSU (~650 base pairs) [51,52], and gGAPDH (~800 bp) [53,54]. Electrophoresis of PCR products were visualized using a 2% agarose gel, stained with GelRed–Biotium and visualized under UV (ultraviolet) light. All reactions included distilled water as a negative control and *T. cruzi* strain SylvioX/10cl1 as a positive control [55].

The positive *Leishmania* sp. kDNA-PCR skin sample was submitted to additional PCR reactions: HSP70 (234 bp) [49,56] and ITS-1 (350bp) [57]. Those reactions were performed according to the original protocols, with few modifications. Protocols were tested with 7 µL of DNA sample employing the Platinum^®^ Taq DNA Polymerase High Fidelity enzyme (Invitrogen, Carlsbad, CA, USA) and, alternatively, GoTaq^®^ DNA Polymerase (Promega, Madison, WI, EUA); besides increasing MgCl_2_ (Magnesium Chloride) concentrations aiming to favor the sensitivity of the reaction. The product of the first reaction (7µl) was also used as a template for a second reaction under the same conditions. Electrophoresis was performed on 8% polyacrylamide gel and silver stained using a proper kit (DNA Silver Staining, GE Healthcare).

We submitted all positive PCR products (except those derived from CP) to purification using DNA purification kit (GE Healthcare Life Sciences, Little Chalfont, Buckinghamshire, UK) following the manufacturer’s instructions. The sequencing reaction was performed using the BigDye Terminator v3.1 Cycle Sequencing kit (Applied Biosystems, Foster City, CA, USA) and applied to the ABI3730 DNA analyzer automatic sequencer (Applied) in the Sequencing Platform (RPT01A) of the Oswaldo Cruz Foundation, RJ. The sequences were assembled and edited using SeqMan (DNASTAR Lasergene, Gatc, Konstanz, Germany) to obtain consensus sequences, which were then aligned and corrected using BioEdit Version 7.0.5.3. The sequences were compared to nucleotide sequences deposited in GenBank (National Center for Biotechnology Information (NCBI)) using the BLAST (Basic Local Alignment Search Tool) algorithm.

### 4.4. Phylogenetic Analysis

The analyzed sequences were merged and aligned in Clustal W (with manual refinement of erroneous readings), by the Mega X program [58]. Phylogenetic tree construction was performed using the online PhyML 3.0 program [59]. We used the ML (Maximum Likelihood) method, employing the best DNA model. The best substitution model was identified as having the lowest BIC score (Bayesian Information Criterion): K80 + G (Kimura, 1980 with G: Gamma distributed rate variation among sites = K2P + G) for the 18S rDNA gene, and GTR + I (General Time Reversible [60], with the fraction of locations that is evolutionarily invariable (+ I)) for the gGAPDH gene, and a bootstrap value of 1000 replicates. We used *T. cruzi* (TcI to TcVI), *T. c. marinkellei, T. erneyi, T. dionisii* and *T. rangeli* sequences from GenBank as references. All sequences analyzed were deposited in the GenBank database, and the accession numbers are represented in Table 2.

### 4.5. Serological Diagnosis

For IgG antibody detection in the sera of wild and domestic canids, an IFAT assay was performed as previously described [61]. Antigens used were the reference strains I00/BR/00F (TcI) and MHOM/BR/1957/Y (TcII) for *T. cruzi* and *L. infantum* (IOC/L579) and *L. braziliensis* (IOC/L566) for *Leishmania* spp. from axenic cultures, mixed in equal (1:1) proportion. Sera from all canids were tested with anti-dog IgG (Immunoglobulin G) conjugated with fluorescein isothiocyanate (Sigma, St. Louis, MO, USA), and the cut-off point adopted was 1/40 [55].

In addition to IFAT, the sera were submitted to ELISA (Enzyme-Linked Immunosorbent Assay) to examine the infection by *T. cruzi* [62] and *Leishmania* sp. (ELISA, Biomanguinhos, Rio de Janeiro (RJ), Brazil) using commercial peroxidase-conjugated antibodies (Sigma, St. Louis, MO, USA). The cut-off point was established by the mean OD (Optical Density) of the negative control ± three standard deviation and the gray range adopted was 20% above the cut-off value. For each serological reaction, two positive and three negative controls for *T. cruzi* and *Leishmania* spp. were added. For the diagnosis of *L. infantum* infection was also performed the TR DPP^®^.

We adopted as seropositivity criteria the positive reaction in two serological tests. We considered mixed infection when there was positivity in two serological tests for both *T. cruzi* and *Leishmania* spp. and the difference between them was less than two serological titers in IFAT assays. We considered *L. infantum* infection when the TR DPP^®^ (BioManguinhos, Rio de Janeiro, RJ, Brazil) was positive, added to another positive serological test for *Leishmania* spp. (IFAT and/or ELISA).

### 4.6. Statistical Analysis

We determined infection rate by the number of mammals that had at least one positive parasitological test and/or two serological tests divided by the total of mammals examined. To define the infected animals, we considered positive results in either parasitological or serological assays in any sampling event, and counted the individual only once, even those recaptured. No animal was examined more than once in the same year; that is, each recapture event occurred in different years (campaigns).

### 4.7. Ethics Statement

The study was approved by the CEUA LW81-12 (Ethics Committee of Foundation Oswaldo Cruz/FIOCRUZ) and CEUA 086/2014 (Ethics Committee of Federal University of Goiás). Captures and samples collection of wild canids were granted by the ICMBio/SISBIO (Brazilian Government Chico Mendes Institute for Conservation of Biodiversity—under license: 14576). All handling procedures with animals followed the American Society of Mastozoology [63].

### 4.8. Construction of the Maps

Traps for capturing wild canids, as well as the headquarters of the farms where samples of the dogs were collected, were georeferenced using GPS (Global Positioning System). These points were visualized in a GIS (Geographic Information System) in the WGS 84 (World Geodetic System). Map construction was performed in QGIS (Quantum GIS software version 3.12), using the continental, national, state, and municipal boundaries of the study area, extracted from the open access cartographic base of IBGE (Brazilian Institute of Geography and Statistics). Google Earth Satellite images (QGIS QuickMapServices plugin) and Collection 4.1—2017 images (QGIS MapbiomasCollection plugin) were also used.

## Figures and Tables

**Table 1:** Wild canids captured (2013–2017) and domestic canids sampled (2014–2016) at Limoeiro Region, municipality of Cumari, Goiás, Brazil.

Canids	Scientific Names	Sampling Events	Number of Individuals	Total of Recaptures	Recaptured Individuals	Individuals Recaptured More Than Once
Domestics	*Canis lupus familiaris*	283	187	94	66	28
Wild	*Cerdocyon thous*	78	72	6	5	1
	*Lycalopex vetulus*	42	29	13	10	3
	*Chrysocyon brachyurus*	11	10	1	1	0
Total wild canids		131	111	20	16	4

**Table 2:** Parasitological and molecular diagnosis of positive hemocultures derived from wild and domestic canids from Cumari, Goiás, Brazil.

Species	Year of Capture	LBT Number	COLTRYP Number	Molecular Target and *T. cruzi* Genotypes		GenBank Sequences Access Number
				18S rDNA	gGAPDH	
*Lycalopex vetulus*	2013	LBT 11477	00537	TcIII/V	TcIII/V/VI	MT705719 MT892929
*Lycalopex vetulus*	2014	LBT 6948	CP	TcIII/V	N.I.	MT678509
*Cerdocyon thous*	2016	LBT 7984	CP	TcIII/V	N.A.	MT678510
*Canis lupus familiaris*	2016	LBT 7202	00723	TcIII/V	TcIII/V/VI	MT705720 MT892930
*Canis lupus familiaris*	2016	LBT 8600	CP	TcIII/V	N.A.	MT678511

COLTRYP, Coleção de *Trypanosoma* de Mamíferos Silvestres, Domésticos e Vetores; COLTRYP/Fiocruz (Oswaldo Cruz Foundation, Rio de Janeiro, RJ/Brazil); 18S rDNA, Small subunit ribosomal DNA; gGAPDH, Glycosomal glyceraldehyde-3-phosphate dehydrogenase; C.P., Culture Pellet; N.I., Not identified; N.A., Not amplified.

**Table 3:** Seropositivity (absolute numbers and percentage) and serological titers (Indirect Immunofluorescence Antibody Test (IFAT)) for *Trypanosoma cruzi, Leishmania* spp. and mixed infection in wild and domestic canids of Cumari, Goiás, Brazil.

Species	Sampling Events	Seropositivity			Serological Titers (IFAT)								
		*Trypanosoma cruzi*	*Leishmania* spp.	Mixed Infection	*Trypanosoma cruzi*					*Leishmania* spp.			
					1/40	1/80	1/160	1/320	1/2560	1/40	1/80	1/160	1/320
*Canis lupus familiaris*	283	31 (11%)	2 * (0.7%)	5 * (1.7%)	20	7	6	1	2	3	3	1	0
*Cerdocyon thous*	78	17 (21.8%)	3 ** (3.8%)	5 (6.4%)	10	8	3	1	0	3	4	0	1
*Lycalopex vetulus*	42	17 (40.5%)	2 (4.7%)	1 (2.4%)	2	4	6	5	0	1	1	1	0
*Chrysocyon brachyurus*	11	2 (18.2%)	0 (0%)	0 (0%)	1	0	1 ***	0	0	0	0	0	0

* *Leishmania infantum* (*N* = 1) and mixed *T. cruzi/Leishmania infantum* (*N* = 2). ** *Leishmania infantum* (*N* = 1). Positive samples also in TR DPP^®^ *** Maned wolf positive in skin culture that it was not possible to recover DNA.

**Table 4:** Temporal serological pattern of *Trypanosoma cruzi* and *Leishmania* spp. in wild and domestic canids throughout captures and recaptures conducted in Cumari, Goiás, Brazil, between 2013 and 2017.

	Year of Expeditions	2013			2014			2015			2016			2017			Total Captured and Recaptured		
	Capture (C) Recapture (R)	C	R	P (%)	C	R	P (%)	C	R	P (%)	C	R	P (%)	C	R	P (%)	C	R	P (%)
*Cerdocyon thous*	N	32	0		6	0		10	2		15	3		9	1		72	6	
	*Trypanosoma cruzi*	10		31%	2		33%	0	1	8%	1	1	11%	2	0	20%	15	2	22%
	*Leishmania* spp.	1		3%	0		0%	0	1 *	8%	1	0	5.5%	0	0	0%	2	1	4%
	Mixed infection	2		6%	0		0%	1	0	8%	1	0	5.5%	1	0	10%	5	0	6.5%
*Lycalopex vetulus*	N	11	0		9	6		2	2		5	3		2	2		29	13	
	*Trypanosoma cruzi*	5		45%	3	4 (2) *	47%	0	2	50%	0	2	25%	0	1*	25%	8	9	40%
	*Leishmania* spp.	1		9%	0	0	0%	0	0	0%	0	0	0%	0	0	0%	1	0	2%
	Mixed infection	1		9%	0	0	0%	0	0	0%	0	0	0%	0	0	0%	1	0	2%
*Chrysocyon brachyurus*	N	3	0		4	0		1	1		2	0		0	0		10	1	
	*Trypanosoma cruzi*	1		33%	1		25%	0	0	0%	0		0%				2	0	18%
	*Leishmania* spp.	0		0%	0		0%	0	0	0%	0		0%				0	0	0%
	Mixed infection	0		0%	0		0%	0	0	0%	0		0%				0	0	0%
*Canis lupus familiaris*	N	0	0		96	0		54	34		37	62		0	0		187	96	
	*Trypanosoma cruzi*				14		14.5%	6	6 (1) *	14%	2	7 (1) *	9%				22	13	12%
	*Leishmania* spp.				1		1%	1	0	1%	0	0	0%				2	0	0.7%
	Mixed infection				0		0%	4	1	6%	0	0	0%				4	1	1.8%

*N* = total number of captured canids; *P* (%) = percentage of infected animals per year. The asterisk (*) refers to seroconversion events.

## References

[1] Lukeš J., Butenko A., Hashimi H., Maslov D.A., Votýpka J., Yurchenko V. (2018). Trypanosomatids Are Much More than Just Trypanosomes: Clues from the Expanded Family Tree. Trends Parasitol..

[2] Roque A.L.R., Jansen A.M. (2014). Wild and synanthropic reservoirs of *Leishmania* species in the Americas. Int. J. Parasitol. Parasites Wildl..

[3] Jansen A.M., Xavier S.C.C., Roque A.L.R. (2015). The multiple and complex and changeable scenarios of the *Trypanosoma cruzi* transmission cycle in the sylvatic environment. Acta Trop..

[4] Sillero-Zubiri C., Wilson D.E., Mittermeierm R.A. (2009). Family *Canidae* (dogs). Handbook of Mammals of the World.

[5] Dalponte J.C. (2009). *Lycalopex vetulus* (*Carnivora: Canidae*). Mamm. Species.

[6] Lemos F.G., Azevedo F.C., Paula R.C., Dalponte J. (2020). *Lycalopex vetulus,* hoary fox. IUCN Red List Threat. Species.

[7] Facure K.G., Giaretta A.A., Monteiro-Filho E.L.A. (2003). Food habits of the crab-eating-fox, *Cerdocyon thous,* in an altitudinal forest of the Mantiqueira Range, southeastern Brazil. Mammalia.

[8] Jácomo A.T.A., Silveira L., Diniz-Filho J.A.F. (2004). Niche separation between the maned wolf (*Chrysocyon brachyurus*), the crab-eating fox (*Dusicyon thous*) and the hoary fox (*Dusicyon vetulus*) in central Brazil. J. Zool..

[9] Kotviski B.M., Facure K.G., de Azevedo F.C., Freitas-Junior M.C., Lemos F.G. (2019). Trophic niche overlap and resource partitioning among wild canids in an anthropized neotropical ecotone. Mastozool. Neotrop..

[10] Rocha F.L., Roque A.L.R., de Lima J.S., Cheida C.C., Lemos F.G., de Azevedo F.C., Arrais R.C., Bilac D., Herrera H.M., Mourão G. (2013). *Trypanosoma cruzi* Infection in Neotropical Wild Carnivores (Mammalia: Carnivora): At the Top of the *T. cruzi* Transmission Chain. PLoS ONE.

[11] Barros J.H.S., Xavier S.C.C., Bilac D., Lima V.S., Dario M.A., Jansen A.M. (2017). Identification of novel mammalian hosts and Brazilian biome geographic distribution of *Trypanosoma cruzi* TcIII and TcIV. Acta Trop..

[12] Reis F.C., Minuzzi-Souza T.T.C., Neiva M., Timbó R.V., Morais I.O.B., Lima T.M., Hecht M., Nitz N., Gurgel-Gonçalves R. (2020). Trypanosomatid infections in captive wild mammals and potential vectors at the Brasilia Zoo, Federal District, Brazil. Vet. Med. Sci..

[13] Courtenay O., Santana E.W., Johnson P.J., Vasconcelos I.A.B., Vasconcelos A.W. (1996). Visceral leishmaniasis in the hoary zorro *Dusicyon vetulus*: A case of mistaken identity. Trans. R. Soc. Trop. Med. Hyg..

[14] Deane L.M., Deane M.P. (1955). Observações preliminares sobre a importância comparativa do homem, do cão e da raposa (*Lycalopex vetulus*) como reservatórios da Leishmania donovani em áreas endêmicas de Calazar, no Ceará. Hospital.

[15] Figueiredo F.B., Gremião I.D.F., Pereira S.A., Fedulo L.P., Menezes R.C., Balthazar D.A., Schubach T.M.P., Madeira M.F. (2008). First report of natural infection of a bush dog (*Speothos venaticus*) with *Leishmania (Leishmania) chagasi* in Brazil. Trans. R. Soc. Trop. Med. Hyg..

[16] Dantas-Torres F. (2009). Canine leishmaniosis in South America. Parasites Vectors.

[17] Crisante G., Rojas A., Teixeira M.M.G., Añez N. (2006). Infected dogs as a risk factor in the transmission of human *Trypanosoma cruzi* infection in western Venezuela. Acta Trop..

[18] Gürtler R.E., Cecere M.C., Lauricella M.A., Cardinal M.V., Kitron U., Cohen J.E. (2007). Domestic dogs and cats as sources of *Trypanosoma cruzi* infection in rural northwestern Argentina. Parasitology.

[19] Kjos S.A., Snowden K.F., Craig T.M., Lewis B., Ronald N., Olson J.K. (2008). Distribution and characterization of canine Chagas disease in Texas. Vet. Parasitol..

[20] Das Xavier S.C.C., Roque A.L.R., dos Lima V.S., Monteiro K.J.L., Otaviano J.C.R., da Silva L.F.C.F., Jansen A.M. (2012). Lower richness of small wild mammal species and chagas disease risk. PLoS Negl. Trop. Dis..

[21] Butler J.R.A., du Toit J.T., Bingham J. (2004). Free-ranging domestic dogs (*Canis familiaris*) as predators and prey in rural Zimbabwe: Threats of competition and disease to large wild carnivores. Biol. Conserv..

[22] Daszak P., Cunningham A.A., Hyatt A.D. (2001). Anthropogenic environmental change and the emergence of infectious diseases in wildlife. Acta Trop..

[23] Kazemi H., Klug H., Kamkar B. (2018). New services and roles of biodiversity in modern agroecosystems: A review. Ecol. Indic..

[24] Moonen A.C., Bàrberi P. (2008). Functional biodiversity: An agroecosystem approach. Agric. Ecosyst. Environ..

[25] Lemos F.G. (2016). Ecologia e Conservação Da Raposa-Do-Campo (Lycalopex vetulus) e Interações com Canídeos Simpátricos em Áreas Antropizadas do Brasil Central.

[26] Bickley S.M., Lemos F.G., Gilmore M.P., Azevedo F.C., Freeman E.W., Songsasen N. (2019). Human perceptions of and interactions with wild canids on cattle ranches in central Brazil. Oryx.

[27] Lemos F.G., Azevedo F.C., Costa H.C.M., Joares A. (2011). Human threats to hoary and crab-eating foxes in central Brazil. Canid News.

[28] Lemos F.G., Azevedo F.C., Beisiegel B.M., Jorge R.P.S., Paula R.C., Rodrigues F.H.G., Rodrigues L.A. (2013). Avaliação do risco de extinção da Raposa-do-campo *Lycalopex vetulus* (Lund, 1842) no Brasil. Biodiversidade Bras..

[29] Correa J.P., Bacigalupo A., Yefi-Quinteros E., Rojo G., Solari A., Cattan P.E., Botto-Mahan C. (2020). Trypanosomatid infections among vertebrates of chile: A systematic review. Pathogens.

[30] Otranto D., Cantacessi C., Pfeffer M., Dantas-Torres F., Brianti E., Deplazes P., Genchi C., Guberti V., Capelli G. (2015). The role of wild canids and felids in spreading parasites to dogs and cats in Europe Part I: Protozoa and tick-borne agents. Vet. Parasitol..

[31] Brandão E.M.V., Xavier S.C.C., Carvalhaes J.G., D’andrea P.S., Lemos F.G., Azevedo F.C., Cássia-Pires R., Jansen A.M., Roque A.L.R. (2019). Trypanosomatids in small mammals of an agroecosystem in central brazil: Another piece in the puzzle of parasite transmission in an anthropogenic landscape. Pathogens.

[32] Marinho-Filho J., Rodrigues F.H.G., Juarez K.M., Oliveira P., Marquis R. (2002). The cerrado mammals: Diversity, ecology, and natural history. The Cerrados of Brazil.

[33] Paglia A.P., Fonseca G.A.B., Rylands A.B., Herrmann G., Aguiar L.M.S., Chiarello A.G., Leite Y.L.R., Costa L.P., Siciliano S., Kierulff M.C.M. (2012). Lista anotada dos mamíferos do brasil/annotated checklist of Brazilian mammals, 2^a^ Edição. Occas. Pap. Conserv. Biol..

[34] Morters M.K., Mckinley T.J., Restif O., Conlan A.J.K., Cleaveland S., Hampson K., Whay H.R., Damriyasa I.M., Wood J.L.N. (2014). The demography of free-roaming dog populations and applications to disease and population control. J. Appl. Ecol..

[35] Sepúlveda M.A., Singer R.S., Silva-Rodriǵuez E., Stowhas P., Pelican K. (2014). Domestic dogs in rural communities around protected areas: Conservation problem or conflict solution?. PLoS ONE.

[36] Vanak A.T., Gompper M.E. (2009). Dogs canis familiaris as carnivores: Their role and function in intraguild competition. Mamm. Rev..

[37] Dürr S., Ward M.P. (2014). Roaming behaviour and home range estimation of domestic dogs in Aboriginal and Torres Strait Islander communities in northern Australia using four different methods. Prev. Vet. Med..

[38] Sepúlveda M.A., Pelican K., Cross P., Eguren A., Singer R.S. (2015). Fine-scale movements of rural free-ranging dogs in conservation areas in the temperate rainforest of the coastal range of southern Chile. Mamm. Biol..

[39] Machado E.M.M., Fernandes A.J., Murta S.M.F., Vitor R.W.A., Camilo Júnior D.J., Pinheiro S.W., Lopes E.R., Adad S.J., Romanha A.J., Dias J.C.P. (2001). A study of experimental reinfection by *Trypanosoma cruzi* in dogs. Am. Soc. Trop. Med. Hyg..

[40] Lemos F.G., Facure K.G., Azevedo F.C., Rosalino L.M., Gheler Costa C.E. (2011). A first approach to the comparative ecology of the hoary fox and the crab-eating fox in a fragmented human altered landscape in the Cerrado Biome at Central Brazil. Middle-Sized Carnivores in Agricultural Landscapes.

[41] Mendes P.C., Beatriz M., de Paula C., Limongi J.E. (2008). Chagas disease and the space distribution of captured triatomines in Uberlândia, Minas Gerais-Brazil. Hygeia.

[42] Spotin A., Parvizi P. (2015). Comparative study of viscerotropic pathogenicity of *Leishmania major* amastigotes and promastigotes based on identification of mitochondrial and nucleus sequences. Parasitol. Res..

[43] Lainson R., Shaw J.J., Silveira F.T., Braga R.R. (1987). American visceral leishmaniasis: On the origin of *Leishmania (Leishmania) chagasi*. Trans. R. Soc. Trop. Med. Hyg..

[44] Courtenay O., Quinnell R.J., Garcez L.M., Dye C. (2002). Low infectiousness of a wildlife host of *Leishmania infantum*: The crab-eating fox is not important for transmission. Parasitology.

[45] Alvares C.A., Stape J.L., Sentelhas P.C., de Moraes Gonçalves J.L., Sparovek G. (2013). Köppen’s climate classification map for Brazil. Meteorol. Z..

[46] Brady C.A., Eisenberg J.F. (1979). Observations on the behavior and ecology of the crab-eating-fox (Cerdocyon thous). Vertebrate Ecology in the Northern Neotropics.

[47] De Campos M.P., da Silva D.A., de Madeira M.F., Velho A.A.M.V., Figueiredo F.B. (2013). First autochthonous case of canine visceral leishmaniasis in Volta Redonda, Rio de Janeiro, Brazil. Rev. Bras. Parasitol. Vet..

[48] Sambrook J., Fritsch E.F., Maniatis T. (1989). Molecular Cloning: A Laboratory Manual.

[49] Cássia-Pires R., Boité M.C., D’Andrea P.S., Herrera H.M., Cupolillo E., Jansen A.M., Roque A.L.R. (2014). Distinct *Leishmania* Species Infecting Wild Caviomorph Rodents (Rodentia: Hystricognathi) from Brazil. PLoS Negl. Trop. Dis..

[50] Degrave W., Fernandes O., Campbell D., Bozza M., Lopes U. (1994). Use of molecular probes and PCR for detection and typing of *Leishmania*-A mini-review. Mem. Inst. Oswaldo Cruz.

[51] Noyes H.A., Stevens J.R., Teixeira M., Phelan J., Holz P. (1999). A nested PCR for the ssrRNA gene detects *Trypanosoma binneyi* in the platypus and *Trypanosoma* sp. in wombats and kangaroos in Australia. Int. J. Parasitol..

[52] Smith A., Clark P., Averis S., Lymbery A.J., Wayne A.F., Morris K.D., Thompson R.A. (2008). Trypanosomes in a declining species of threatened Australian marsupial, the brush-tailed bettong *Bettongia penicillata* (Marsupialia: *Potoroidae*). Parasitology.

[53] Borghesan T.C., Ferreira R.C., Takata C.S.A., Campaner M., Borda C.C., Paiva F., Milder R.V., Teixeira M.M.G., Camargo E.P. (2013). Molecular phylogenetic redefinition of *Herpetomonas* (*Kinetoplastea, Trypanosomatidae*), a genus of insect parasites associated with flies. Protist.

[54] Hamilton P.B., Adams E.R., Njiokou F., Gibson W.C., Cuny G., Herder S. (2009). Phylogenetic analysis reveals the presence of the *Trypanosoma cruzi* clade in African terrestrial mammals. Infect. Genet. Evol..

[55] Dario M.A., Lisboa C.V., Costa L.M., Moratelli R., Nascimento M.P., Costa L.P., Reis Leite Y.L., Llewellyn M.S., das Chagas Xavier S.C., Rodrigues Roque A.L. (2017). High *Trypanosoma* spp. diversity is maintained by bats and triatomines in Espírito Santo state, Brazil. PLoS ONE.

[56] Graça G.C., Volpini A.C., Romero G.A.S., Neto M.P., de Hueb M.O., Porrozzi R., Boité M.C., Cupolillo E. (2012). Development and validation of PCR-based assays for diagnosis of American cutaneous leishmaniasis and identification of the parasite species. Mem. Inst. Oswaldo Cruz.

[57] Schönian G., Nasereddin A., Dinse N., Schweynoch C., Schallig H.D.F.H., Presber W., Jaffe C.L. (2003). PCR diagnosis and characterization of *Leishmania* in local and imported clinical samples. Diagn. Microbiol. Infect. Dis..

[58] Kumar S., Stecher G., Li M., Knyaz C., Tamura K. (2018). MEGA X: Molecular evolutionary genetics analysis across computing platforms. Mol. Biol. Evol..

[59] Guindon S., Dufayard J.F., Lefort V., Anisimova M., Hordijk W., Gascuel O. (2010). New algorithms and methods to estimate maximum-likelihood phylogenies: Assessing the performance of PhyML 3.0. Syst. Biol..

[60] Tavaré S. (1986). Some probabilistic and statistical problems in the analysis of DNA sequences. Am. Math. Soc. Lect. Math. Life Sci..

[61] Camargo E.M. (1966). Fluorescent antibody test for the serodiagnosis of American Trypanosomiasis. Technical modification employing preserved culture forms of *Trypanosoma cruzi* in a slide test. Rev. Inst. Med. Trop. São Paulo.

[62] Dario M.A., Rodrigues M.S., Barros J.H.D.S., Xavier S.C.D.C., D’Andrea P.S., Roque A.L.R., Jansen A.M. (2016). Ecological scenario and *Trypanosoma* cruzi DTU characterization of a fatal acute Chagas disease case transmitted orally (Espírito Santo state, Brazil). Parasites Vectors.

[63] Sikes R.S., Gannon W.L. (2011). Guidelines of the American Society of Mammalogists for the use of wild mammals in research. J. Mammal..

